# A case of successful coronary angioplasty in an achondroplasia patient with total occlusion of an anomalous right coronary artery (case report)

**DOI:** 10.1186/s12872-020-01612-z

**Published:** 2020-07-10

**Authors:** Mustafa Ibrahiem Mohammed Ali, Ahmed Abdalazim Dafallah Albashir, Omer Ali Mohamed Ahmed Elawad, Makram Aboali Ebied Mohamed

**Affiliations:** 1 Department of Internal Medicine, Faculty of Medicine, University of Gezira, Wad Medani, Sudan; 2 Teaching assistant, Faculty of Medicine, University of Gezira, Wad Medani, Sudan; 3 Resident, Wad Medani Heart Centre, Wad Medani, Sudan; 4 Khartoum North University, Wad Medani, Sudan

**Keywords:** Achondroplasia, Percutaneous coronary intervention, Coronary arteries, Anomalies

## Abstract

**Background:**

Coronary interventions in patients of achondroplasia have been reported rarely in the medical literature. Due to short stature and kyphoscoliosis, endovascular access (Cannulation) of the coronary arteries is usually extremely difficult in such patients.

**Case presentation:**

A 33 years old patient, a known case of achondroplasia, presented with epigastric pain for 3 h duration to a university hospital, Sudan. Her height was 95 cm and her weight was 38 Kg. A trans-femoral approach for coronary angioplasty was preferred. After it has been extremely difficult to cannulate the left system at first, the cannulation has been performed successfully using 5F, JL3.5 catheter. The angiogram depicted total occlusion of the proximal right coronary artery which was found to be originating from the left coronary sinus of the aorta. Successful trans-femoral coronary angioplasty has been performed with stent placement, and no complications encountered. During her last follow up, 1 year after the procedure, she appeared to be free of symptoms and with no further ischemic attacks or procedure-related complications.

**Conclusions:**

To the best of our knowledge, this is the first reported case of successful coronary angioplasty in achondroplasia patient in whom the occluded artery is an anomalous coronary artery. Literature review, description of the achondroplasia, development of the coronary arteries and the hypothesized theory for the anomaly have been described in this case report. The PCI performed has also been clearly and comprehensively described.

## Background

Achondroplasia is the commonest human bone dysplasia. Its prevalence is approximately 1 in 20,000 live births [1]. It has an autosomal dominant pattern of inheritance, and caused by mutations in the fibroblast growth factor receptor 3 (FGFR3) gene. The most notable clinical features include disproportionate short stature (adult height is approximately 4 ft), rhizomelic shortening (shortening of the long bones in which the proximal segments more severely affected than the distal segments), and macrocephaly [2]. Achondroplasia affects motor development early on but has no impact on cognition. The average life expectancy is decreased by 10 years [3].

The overall heart disease mortality rate is over two-fold greater in patients with achondroplasia more than that of the general population [3]. Although heart diseases are quite common in achondroplasia patients, there are limited data on the optimal approach regarding coronary interventions. Furthermore, and to the best of authors’ knowledge, there are no reported cases in the medical literature regarding the coronary angioplasty in those suffering from achondroplasia who have an anomaly in the coronary circulation.

The incidence of coronary artery anomalies detected by coronary angiography is about 1.3% in the largest reported series [4]. Anomalous origin of right coronary artery (RCA) from the left sinus is a rare congenital anomaly that occurs in 0.05–0.1% of the general population. Right coronary artery’s anomalous origin is more frequent than left coronary artery (LCA) but the Sudden cardiac death (SCD) is more frequent with the anomalous LCA.

Most patients are asymptomatic but may experience ischemic symptoms, arrhythmias or even sudden cardiac death.

Herewith, we report an interesting case of achondroplasia in whom 5F, JL3.5 catheter has been successfully used for the coronary angioplasty. During the angiogram, the RCA was found to be originating from the left coronary sinus of the aorta with total occlusion of the proximal RCA. The patient underwent successful trans-femoral coronary angioplasty and stent placement with no complications.

## Case presentation

A 33 years old Sudanese woman with a prominent short stature came to a university hospital, Sudan, with her family after developing epigastric pain for 3 h duration. There was no history of diabetes, hypertension or dyslipidaemia. She has been previously diagnosed with achondroplasia.

**Fig. 1:**
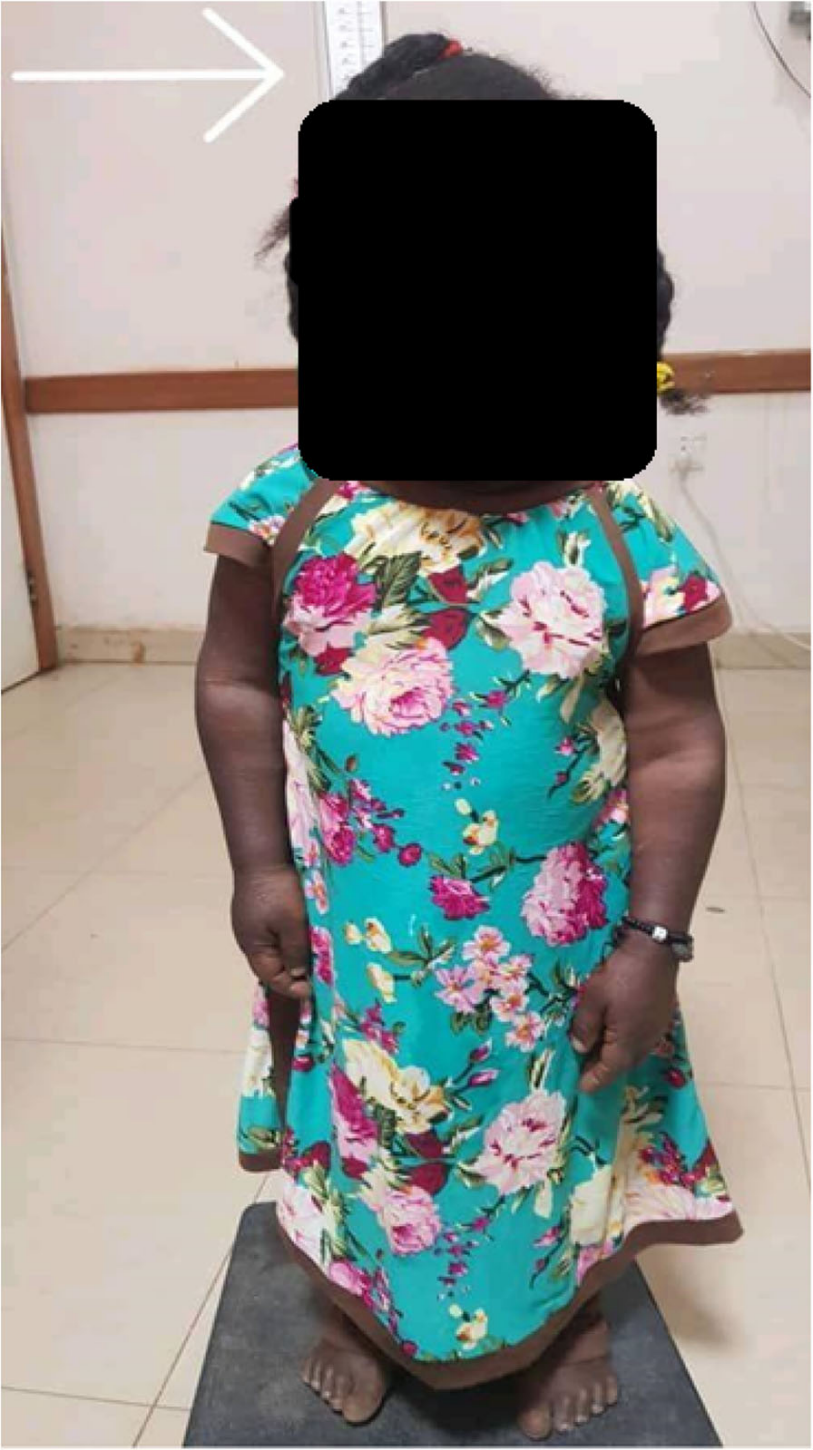
Clinical picture of patient showing short stature and rhizomelic shortening of arms and legs

Physical examination revealed that the patient looked ill, in pain, and with profuse sweating. Her BP was 100/60, pulse rate 72 regular, oxygen saturation 92% on room air. Her height was 95 cm, her weight was 38 Kg. The extremities have shortening that is more pronounced in the proximal segments, with kyphoscoliosis in her back (Fig. 1). Precordial and respiratory system examinations were normal. Blood samples have been taken for investigations and the results showed quantitative serum troponin of 12.9 ng/ml (Normal lab value 0–0.6 ng/ml), HB was 11 g/dl, blood urea was 11 mg/dl and the serum creatinine of 0.8 mg/dl (Table 1).

**Table 1:** The table describes the results of the blood investigations obtained for the patient

Quantitative serum troponin	12.9 ng/ml (Normal lab value 0-0.6 ng/ml)
HB	10 g / dl
TWBCS	12.3
Platelets	319
RBS	170 mg / 100 ml
HbA1c	5.6 %
INR	1.3
ICT ( HIV,HBV,HCV)	negative
Blood urea	16 mg / dl
Serum creatinine	0.8 mg / dl

**Fig. 2:**
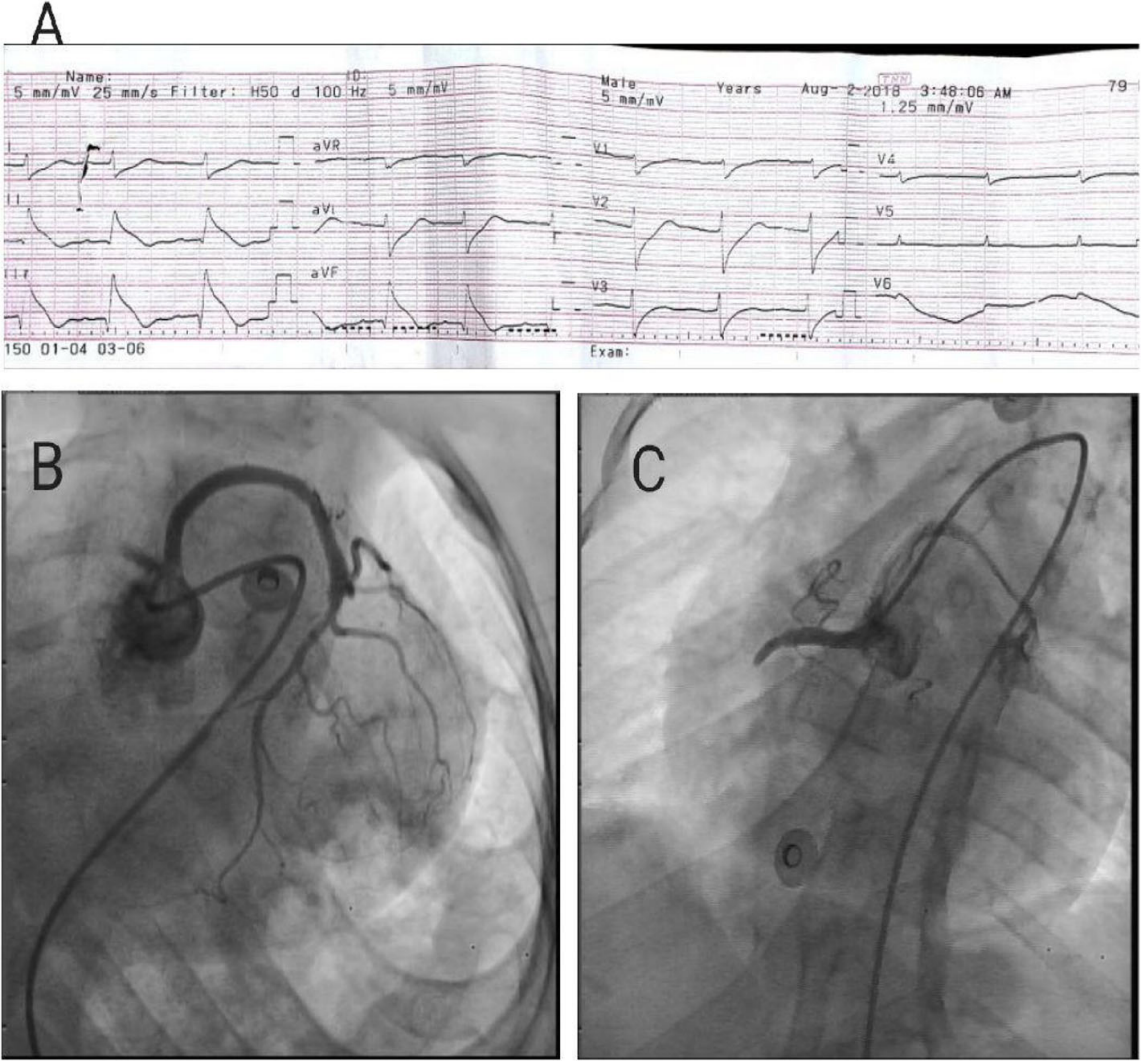
**a** ECG showing ST segment elevation in lead II, III, AVF with reciprocal changes in leads 1 and aVL which suggested acute inferior STEMI. **b** Coronary angiography showing both Left main stem and RCA originating from the left coronary sinus. **c** Coronary angiography showing total occlusion of the proximal RCA

Electrocardiogram showed ST-segment elevation in lead II, III, AFV with reciprocal changes in leads 1 and aVL which suggested acute inferior ST-elevation myocardial infarction (STEMI) (Fig. 2a). She was diagnosed as acute inferior STEMI after 10 min of her arrival. So the time from first medical contact (FMC) to ECG and diagnosis was 10 min. The estimated delay from STEMI diagnosis to primary PCI was less than 120 min. Consequently, primary PCI has been decided according to the European Society of Cardiology (ESC) guidelines. The final diagnosis was inferior STEMI- ACS. Echocardiogram revealed inferior and infero-septal walls hypokinesia with LVEF of 40% and mild mitral regurgitation, normal other valves. She received Aspirin, Clopidogrel, and the patient was taken immediately for primary percutaneous coronary intervention (PCI). The consent has been obtained from her mother after explaining potential risks and safeguards of the procedure. Each one of the previous medical decisions was made according to the ESC guidelines (https://www.escardio.org/Guidelines/Clinical-Practice-Guidelines/ESC-EACTS Guidelines-in-Myocardial-Revascularisation-Guidelines-for).

**Fig. 3:**
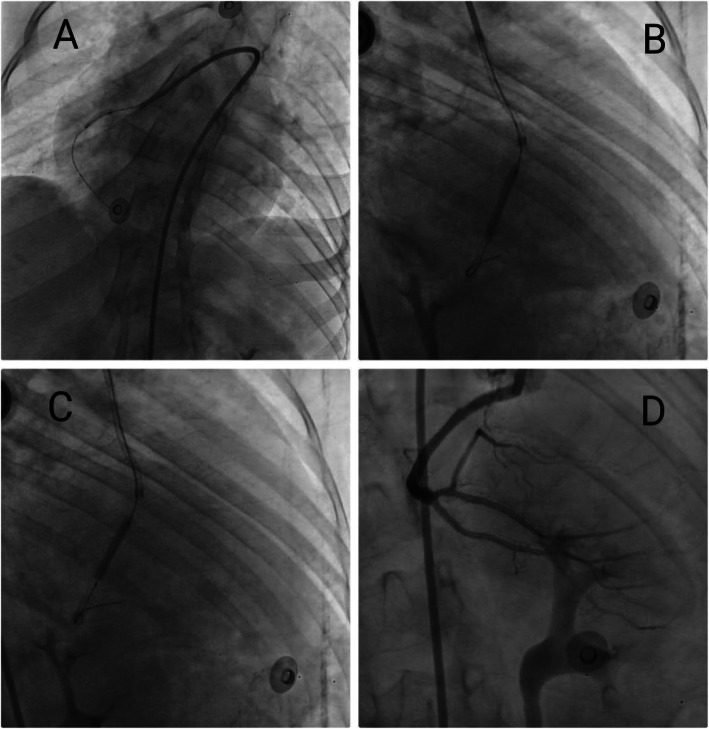
**a** Coronary angiography showing balloon dilatation. **b** Coronary angiography showing proximal stent deployment. **c** Coronary angiography showing distal stent deployment and overlap with proximal stent. **d** Final agiographic result with TIMI flow

Before coronary angiography, right femoral artery approach was chosen regarding the short limbs, elbow angles, and kyphoscoliosis which make the radial approach more challenging. Initially, femoral artery sheath length was compared to the lower limb length to ensure that the limb was long enough to accommodate the femoral sheath. A 5F femoral artery sheath was introduced with keeping the major portions of the catheter and the guide-wire outside the body. Then, a peripheral angiogram was taken to delineate the anatomy of the ileo-femoral system. A guidewire was then passed all the way to the heart in a retrograde fashion. Coronary angiography was done using 5F, JL3.5 catheter although it has been extremely difficult at first to cannulate the left system. The angiogram revealed that the left main stem was long, tortuous and patent. The left anterior descending and left circumflex were normal and patent arteries. There was retrograde filling to RCA. We noticed that the RCA was originating from the left coronary sinus (Fig. 2b). The course of RCA was non-fatal course and the RCA was not vulnerable to be entrapped between the aorta and pulmonary as shown in video 1 (A high-quality fluoroscopy/angiography video is attached). We manipulated the 5F, JL3.5 catheter with gentle rotation to cannulate the RCA which was very easy to engage. The angiogram revealed a total occlusion of the proximal RCA (The culprit vessel) (Fig. 2c). We decided to proceed for PCI using guide catheter XB –RCA with easy engagement. Then, we have used an intermediate support PCI guide-wire (Bostom) with UFH (3.500 IU intravenously). The lesion was pre-dilated using 2 × 12 mm semi-compliant balloon (Bostom), single inflation up to 10 atm (Fig. 3a). Drug-eluting stent 2.75 × 20 mm (Promus element plus) was deployed in the distal pre bifurcation RCA segment and inflated up to 14 ATM. Another drug-eluting stent 2.75 × 18 mm (Orsiro) was deployed in the proximal RCA segment and overlapped with the distal stent and inflated up to 13 ATM (Fig. 3b and c). The suitable stent’ diameter was estimated by comparing the proximal healthy part of the artery with the diameter of the guiding catheter. The overlapped area was dilated with balloon 2.75 × 18 mm, inflated up to 15 ATM with good final angiographic results with TIMI flow III (Fig. 3d). No complications encountered. The patient underwent primary PCI of the culprit vessel (RCA), with the whole procedure has taken about 45 min and the total amount of contrast used was about 200 ml including aortography (A high-quality fluoroscopy/angiography video attached).

**Fig. 4:**
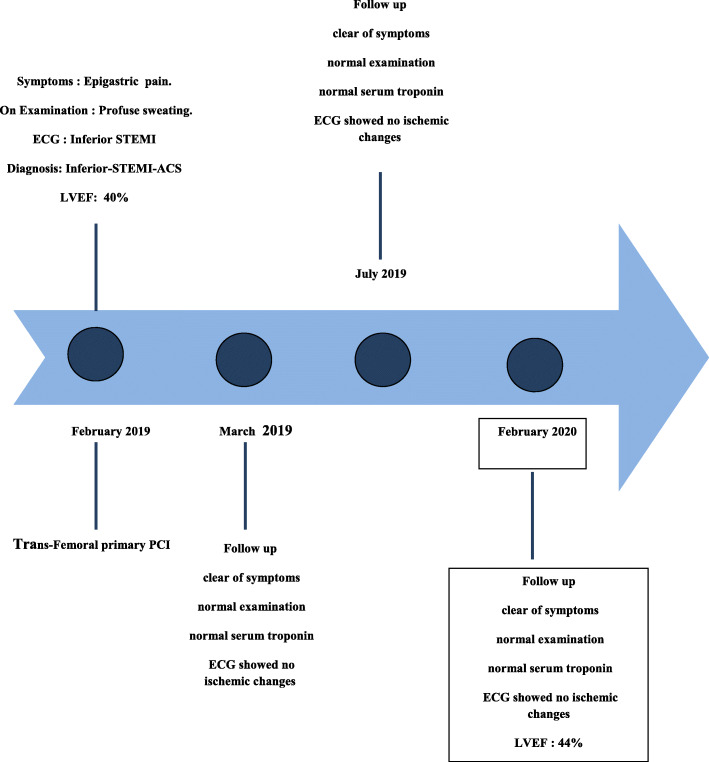
Time line of the events

The course in the hospital was uneventful and the patient was discharged in a stable condition on the third day of hospitalization. At discharge, the patient was put on Aspirin, Clopidogrel, Atorvastatin, and beta-blocker. During her last follow up, 1 year after the PCI, she has been free of symptoms, the ECG was normal and the LVEF was 44%. Clopidogrel has been withdrawn from the medications in the last follow up. Timeline of the events has been shown in Fig. 4.

## Discussion and conclusion

Coronary circulation anomalies result from processes that disrupt the normal differentiation and specialization of the primitive heart tube [5]. Position of the endothelial buds or septation of the truncus arteriosus may give rise to anomalous origins of coronary arteries [6]. The majority of anomalies are discovered incidentally during coronary angiography.

According to the latest guidelines of the European Cardiology Society (ESC), primary PCI is the preferred coronary reperfusion therapy in patients with STEMI, given that the onset of symptoms is up to 12 h ago and provided that the PCI can be delivered within a specific timeframe (up to 2 h) [7]. When conducted in high volume centres, it results in lower mortality. As so, our patient underwent primary PCI in favour of thrombolytic therapy. However, there is insufficient evidence to determine whether primary PCI is still superior outside the specified time frame. In situations where primary PCI can not be performed in a timely fashion, fibrinolysis should be administered as soon as possible, as long as it is up to 12 h from the onset of symptoms and no contraindications to thrombolysis exist. If first medical contact (FMC) was out-of-hospital, pre-hospital thrombolysis (e.g., in an ambulance) should not be delayed. This should be accompanied by transfer to PCI centres for coronary angiography in all patients (https://www.escardio.org/Guidelines/Clinical-Practice-Guidelines/ESC-EACTS Guidelines-in-Myocardial-Revascularisation-Guidelines-for).

Emergency CABG may be indicated in specific STEMI patients who are unfit for PCI. The predicted surgical mortality, the anatomical complexity of CAD, and the anticipated completeness of revascularization are essential criteria for decision-making concerning the type of revascularization (CABG or PCI). Whether conservative therapy, PCI, or CABG is preferred, this should rely on the benefit-risk ratios of these treatment strategies. The risks of periprocedural complications (e.g. new-onset arrhythmias, cerebrovascular events, renal failure, or wound infections) should be weighed against improvements in health-related quality of life and the lasting freedom from repeat revascularization, MI, or death. Although heart diseases occur with increased prevalence in achondroplasia patients, the optimal approach for coronary angioplasty in such patients is not known due to limited data in the medical literature. There have been only four reported cases in the medical literature regarding successful coronary angioplasty in achondroplasia patients. In one similar reported case, the femoral approach was used via a femoral artery sheath [8]. The patient had chronic total occlusion (CTO) of the proximal right coronary artery, which was then cannulated using 6 Fr, 3.5 curves Judkins right guiding catheter. In another achondroplasia patient, anterior ST-segment myocardial infarction (STEMI) was diagnosed and the patient underwent trans-radial sheath-less coronary angiography. Trans radial route was preferred regarding the patient’s underlying interstitial lung disease and to mobilize him early [9]. Engagement of the RCA was performed using a 6 Fr, 4 curve Judkins right (JR4) catheter while VL 3.0 guide was used to engage the left system [9]. Trans-radial coronary angioplasty was also performed in another case [10]. A multi-vessel percutanous trans-luminal coronary angioplasty (PTCA) in achondroplasia patient has been reported in a 46-year-old male with achondroplasia. Initially, Coronary Angiography (CAG) was planned and the radial artery route was used given femoral artery access issues and to avoid local bleeding complication. Surgical revascularization was the initial plan, however, as the CABG required multiple grafts but given severe musculoskeletal deformity, surgeons were not optimistic of suitable grafts. So PTCA was planned via right femoral artery access under Ultrasound guidance. Cannulation of the RCA was performed using 7 Fr, 3.5 curve Judkins right guiding catheter and the LCA was cannulated using 7 Fr, 3.5 curve EBU guiding catheter [11]. There are few reports of coronary artery bypass surgery in this group of patients [12, 13].

In general, away from achondroplasia, radial access for primary PCI was associated with lower risks of access site bleeding, vascular complications, and need for transfusion. Besides, there was a significant mortality benefit in patients allocated to the trans-radial access site. With respect to PCI in patients with achondroplasia, technical difficulties were noted regarding the endovascular access of the coronary arteries using the trans-radial approach due to short limbs, elbow angles, and kyphoscoliosis [10]. Femoral access is also challenging as well, with respect to femoral artery access issues and local bleeding complications, and ultra-sonographic guidance may be required [8, 11].

To the best of our knowledge, this is the first reported case of successful coronary angioplasty in achondroplasia patient in whom the occluded artery is an anomalous coronary artery. The RCA which was found to be originating from the left coronary sinus of the aorta. The report consolidated the previous reports that the JL3.5 catheter can safely be used to perform an angioplasty in achondroplastic patients. Due to the patient’s short stature and kyphoscoliosis, when we first attempted to cannulate the left system, we experienced technical difficulties, but the cannulation has been performed successfully using the JL3.5 catheter. No complications have been noticed.

In conclusion, percutaneous coronary interventions can be performed safely in patients with achondroplasia, even in those with an anomalous coronary artery. Herewith, we have reported a successful trans-femoral coronary angioplasty in achondroplasia patient with total occlusion of the right coronary artery, using the 5F, JL3.5 catheter. Further studies are required to substantiate our report and elucidate the best type of coronary catheters and the optimal approach of coronary angioplasty in achondroplasia patients. Finally, further training for interventionists and catheter-lab technicians in this regard will be of great value to save the life of these subgroup of patients.

## Supplementary information

**Additional file 1: Video 1.** The angiography video.

## Data Availability

The data used in this report is available to readers.

## Abbreviations

IWMI: Inferior wall myocardial infarction
RCA: Right coronary artery
LCA: Left coronary artery
SCD: Sudden cardiac death
ESC: European society of cardiology
CTO: Chronic total occlusion
LVEF: Left ventricular ejection fraction
PCI: Percutaneous coronary intervention
CABG: Coronary artery bypass graft
STEMI: ST-elevation myocardial infarction
CAG: Coronary angiography

## References

[1] Shiang R, Thompson LM, Zhu YZ (1994). Mutations in the transmembrane domain of FGFR3 cause the most common genetic form of dwarfism, achondroplasia. Cell.

[2] Horton WA, Hall JG, Hecht JT (2007). Achondroplasia. Lancet.

[3] Wynn J, King TM, Gambello MJ, Waller DK, Hecht JT (2007). Mortality in achondroplasia study: a 42-year follow-up. Am J Med Genet.

[4] Yamanaka O, Hobbs RE (1990). Coronary artery anomalies in 126,595 patients undergoing coronary arteriography. Catheter Cardiovasc Diagn.

[5] Fitzgerald MJT (1980). Embriologia Humana.

[6] Neufeld HN, Schneeweiss A (1983). Coronary artery disease in infants and children.

[7] West RM, Cattle BA, Bouyssie M, Squire I, de Belder M, Fox KA, Boyle R, McLenachan JM, Batin PD, Greenwood DC, Gale CP (2011). Impact of hospital proportion and volume on primary percutaneous coronary intervention performance in England and Wales. Eur Heart J.

[8] Srinivas SK, Ramalingam R, Manjunath CN (2013). A rare case of percutaneous coronary intervention in achondroplasia. J Invasive Cardiol.

[9] Rahman N, Nabi A, Gul I (2015). Sheathless transradial coronary angioplasty in an achondroplasic patient with ST elevation myocardial infarction. BMJ Case Rep.

[10] Verma B, Singh A, Saxena AK, Kumar M (2018). Transradial coronary angioplasty in an achondroplastic patient with chronic total occlusion (CTO): first case report. J Cardiovasc Dis Res.

[11] Kumar V, Kumar V (2017). A case of multivessel PTCA in achondroplasia patient. Egypt Heart J.

[12] Balaquer JM, Perry D, Crowley J, Moran JM (1995). Coronary artery bypass grafting in an achondroplastic dwarf. Tex Heart Inst J.

[13] Tagarakis GI, Karangelis D, Baddour AJ (2010). Coronary artery surgery in a man with achondroplasia: a case report. J Med Case Rep.

